# Patient-Centered Tablet Application for Improving Medication Adherence after a Drug-Eluting Stent

**DOI:** 10.3389/fpubh.2016.00272

**Published:** 2016-12-12

**Authors:** Vicki Shah, Anandu Dileep, Carolyn Dickens, Vicki Groo, Betty Welland, Jerry Field, Matthew Baumann, Jose D. Flores, Adhir Shroff, Zhongsheng Zhao, Yingwei Yao, Diana J. Wilkie, Andrew D. Boyd

**Affiliations:** ^1^Department of Biomedical and Health Information Sciences, College of Applied Health Sciences, University of Illinois at Chicago, Chicago, IL, USA; ^2^Department of Biobehavioral Health Science, College of Nursing, University of Illinois at Chicago, Chicago, IL, USA; ^3^Division of Cardiology, Department of Internal Medicine, College of Medicine, University of Illinois at Chicago, Chicago, IL, USA; ^4^Department of Pharmacy Practice, College of Pharmacy, University of Illinois at Chicago, Chicago, IL, USA; ^5^University of Illinois at Chicago, Chicago, IL, USA; ^6^Department of Biobehavioral Nursing Science, University of Florida, Gainesville, FL, USA

**Keywords:** mobile health, patient-centered research, health informatics, Kolb learning theory, consumer health informatics

## Abstract

**Background/aims:**

This study’s objective was to evaluate a patient-centered educational electronic tablet application, “My Interventional Drug-Eluting Stent Educational App” (MyIDEA) to see if there was an increase in patient knowledge about dual antiplatelet therapy (DAPT) and medication possession ratio (MPR) compared to treatment as usual.

**Methods:**

In a pilot project, 24 elderly (≥50 years old) research participants were recruited after a drug-eluting stent. Eleven were randomized to the control arm and 13 to the interventional arm. All the participants completed psychological and knowledge questionnaires. Adherence was assessed through MPR, which was calculated at 3 months for all participants who were scheduled for second and third follow-up visits.

**Results:**

Relative to control, the interventional group had a 10% average increase in MPR. As compared to the interventional group, more patients in the control group had poor adherence (<80% MPR). The psychological data revealed a single imbalance in anxiety between the control and interventional groups. On average, interventional participants spent 21 min using MyIDEA.

**Discussion:**

Consumer health informatics has enabled us to engage patients with their health data using novel methods. Consumer health technology needs to focus more on patient knowledge and engagement to improve long-term health. MyIDEA takes a unique approach in targeting DAPT from the onset.

**Conclusion:**

MyIDEA leverages patient-centered information with clinical care and the electronic health record highlighting the patients’ role as a team member in their own health care. The patients think critically about adverse events and how to solve issues before leaving the hospital.

## Introduction

Patient antithrombotic medication adherence following placement of a drug-eluting stent (DES) is a challenge as 5.4% of patients never fill a prescription and on average, patients have their medication only 81% of the time over a year (1). Non-adherence to antithrombotic medication leads to a nine times greater risk of death within the first year of the stent placement (2–5). Failure to adhere to the medication regimen for DES is associated with an increase of 20–40% in mortality rate (3, 4, 6, 7). Secondary prevention of future disease and disability requires patients understanding about the importance of medication adherence; non-adherence is often due to frequent miscommunication between the medical staff and patients regarding the purpose of the medication or the specific duration of medication (8, 9).

This gap indicates that health-care education research is needed to improve patient-centered medical care (2, 8). We aimed to address this gap by creating an educational application that would allow for patient centeredness by involving both patients and medical partners in building the program. Patient-centered outcomes research (PCOR) is used to improve patient education and treatment (10). PCOR focuses on gaps in knowledge and variation in patient care (10). Our study adhered to PCOR methodology standards: (1) we prioritized the research questions, (2) used an appropriate study design, (3) used patient perspectives, and (4) fostered efficient dissemination of the results (11).

While PCOR is critical to improve patient health, learning theories can help structure education to the learning needs of patients while being mindful of their learning styles (11). Kolb’s experiential learning theory is a four part cyclical model addressing four types of learning styles: converger, diverger, assimilator, and accommodator. Kolb’s four-stage learning cycle shows how experience is translated into concepts (12). The four stages include concrete experience, reflective observations, abstract conceptualization, and active experimentation (12). Kolb’s model has been successfully applied to develop a number of patient, medical, and nursing education materials (13–20). PCOR can be improved by mobile health technology (MHT), which has been shown to lower health-care costs and improve patient’s health (21, 22). MHT has been used extensively in studies of diabetes and other chronic diseases (23–31). A few studies have evaluated usage of cell phone reminders and online learning modules with differing results (24, 27, 29).

My Interventional Drug-Eluting Stent Education App (MyIDEA) was developed with a participatory design to provide a user centered educational intervention (32). This electronic tablet application is tailored to patients by utilizing personalized data from their coronary revascularization procedure. Patient’s symptoms are integrated in the application. MyIDEA then prompts the patient to respond to how their symptoms have affected their lives ensuring reflective observation. The aim of this study was to test feasibility and recruitment and to see if there is any positive effect on medication possession ratio (MPR).

## Materials and Methods

A pilot study used a randomized control trial to measure a change in patient antithrombotic medication adherence. The study was conducted at the University of Illinois Hospital and Health Sciences System (UIH) and approved by the IRB. Participants were eligible if they met the following criteria (1) had a percutaneous coronary intervention with a DES at UIH, (2) understood English, and (3) were at least 50 years old (due to funding agency requirement). Exclusion criteria included an allergy to aspirin, thienopyridines (clopidogrel/ticagrelor/prasugrel), and the inability to give informed consent. All subjects gave informed consent with the recommendations of the Belmont Report as approved by the University of Illinois Institutional Review Board (IRB).

The eligible patients were approached 2–4 h after placement of the DES and consented after understanding the risks and benefits. The research nurses administered the Rapid Estimate of Adult Literacy in Medicine-Short Form (REALM-SF) (33) to the consented participants to measure their health literacy, the 36-Item Short Form Health Survey (SF-36) (34), Hospital Anxiety and Depression Scale (HADS) (35), Burden–benefit questionnaire (36), and PCI knowledge questionnaire. The purpose of the psychometric data was to compare the two groups. Randomization occurred upon enrollment, and the psychometrics will reveal if the two groups were randomized equally on common psychometric factors affecting medication adherence. Next, the participants were randomized to either usual care (control arm) or MyIDEA (intervention arm). The participants in the interventional arm completed the educational program while the control arm received usual care that included informal education and used the tablet to play games of checkers and tic-tac-toe. All research participants were scheduled for a second appointment with the research team at their follow-up visit with cardiology following the procedure. The interventional group again interacted with the MyIDEA program and all research participants completed the SF-36, HADS, and PCI knowledge questionnaire a second time. A third visit with the research team was scheduled at 3 months after their first visit date to complete the surveys. At all three visits, a blood draw was performed to measure aspirin reactive unit (ARU) and P2Y12 reaction unit (PRU). The blood tests measured platelet reactivity of both the Aspirin pathway and the P2Y12 protein. A company called VerifyNow produces two tests that measure the ARU and thienopyridine inhibitors, and it is measured as PRU (37). The research participants consented to have their pharmacies contacted to see when and how many prescriptions were filled for a thienopyridine inhibitor of the P2Y12 receptor.

A measure used to evaluate effectiveness of the tablet-based application was MPR. MPR is defined as the percentage of time that a patient has access to medication (38). It is calculated as the ratio of the research participant’s days’ supply of a specific medication to the number of days in the set time period. A MPR of 100% means the patient has enough medication to follow the desired prescription. Any value greater than or equal to 80% reflects good adherence (39). MPR was calculated from the day of consent to 90 days after enrollment. Some research participants were previously on a thienopyridine inhibitor, so refill dates less than 30 days before enrollment were included in the calculation of MPR. Comparison of the MPR difference between the drop out participants and those who attended visits 2 and 3 was conducted. Another measure to evaluate medication adherence involved blood tests of the platelet reactivity of both the ARU and PRU (37). These tests measure the effects of antiplatelet activity on two specific pathways separately. The equations used for obtaining this score were:

- ARUXPRU score = (ARU calculation × PRU calculation);
- ARU calculation = (ARU upper reference value − follow-up ARU)/(ARU upper reference value − ARU floor);
- PRU calculation = (PRU upper reference value − follow-up PRU)/(PRU upper reference value − PRU floor).

**Figure 1:**
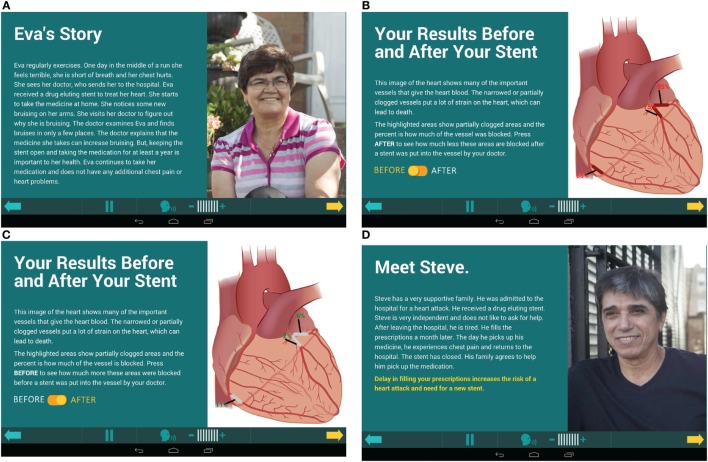
**Images from the MyIDEA Educational application**.

The MyIDEA application taught the importance of adherence to medication. As part of the NCDR Cath PCI 4.4^1^ reporting template, patient symptoms such as heart attack, unstable angina, stable angina, shortness of breath, and fatigue were recorded. They were then taken from the report and tailored to the patient in MyIDEA. Another area of tailoring was to show the patients’ stent placement, a screen with information about the patients’ cardiologist and artery blockage pre- and post-op (Figure 1C) as well as his/her prescription (Figure 1B). By doing this, the patients were able to learn about their procedure and how the DES worked with the antithrombotic medication.

The program integrated active learning to engage patients to think critically about circumstances that could be detrimental to their drug adherence by using five patient stories designed to highlight peer reviewed causes of poor adherence (Figures 1A,D). Each story portrayed a real life situation to which the participants could relate by focusing on common challenges to adherence. They had themes such as monetary restrictions, family living too far away, outside influences altering their medication regime, and side effects of the medication. The patients answered questions regarding the scenarios, which actively engaged them in Kolb’s experiential learning theory. There was use of concrete examples with the patient stories. Also, participants used reflective observation and chose which story they most related to. Abstract conceptualization was used to learn about the importance of DES, what the medication did, why medication was needed daily for a year, and medication adherence. Last, active experimentation about how other patients overcame relatable obstacles helped patients apply the information to themselves.

The MyIDEA program assigned a time stamp to every click in the application as the participants advanced through the program; therefore, the amount of time spent on each of the screens by each participant could be calculated. The program was run listening to the audio of each screen to measure a total time to view the program without additional time for replays. A subgroup analysis was calculated to find the average percent of time spent by all the participants per screen during visit 1 and 2 (Figures 1A–D). Screens 14–16, 18, and 19 are focused on patient-centered data and audio recordings and screens 34–38 are the patient stories.

A multidisciplinary team and patient advocates conducted the analysis of the initial findings and engaged in a discussion about the results.

## Results

Twenty-four participants were recruited and 13 were randomized to the intervention. Thirty declined participation and 50 were ruled ineligible. The participants in this study were on average 60.5 years old (SD 7.0 years) (see Table 1). Of the participants, 58% were males and 42% were females. The grade reading level of the participants was 4% 4th–6th grade, 50% at 7th–8th grade, and 46% greater than 9th grade *via* REALM-SF (33). Of the participants, 63% were African-American, 25% White, 8% Asian, 4% were categorized as more than one race, and 13% were of Hispanic ethnicity. The 75% of participants (73% of control and 77% of MyIDEA) had medication assistance (friends or family members who could help pickup the prescriptions).

**Table 1:** **Demographics of the research participants, with age, gender, race, ethnicity, and reading level**.

Demographics		All	Control	My Interventional Drug-Eluting Stent Educational App	*P*-value
Age	Mean (SD)	60.5 (7.0)	60.6 (4.1)	60.5 (8.9)	1
Gender	Male	14 (58%)	7 (64%)	7 (54%)	0.70
	Female	10 (42%)	4 (36%)	6 (46%)	
Race	White	6 (25%)	3 (27%)	3 (23%)	1
	Black	15 (63%)	7 (64%)	8 (62%)	
	Asian	2 (8%)	1 (9%)	1 (8%)	
	Multirace	1 (4%)	0 (0%)	1 (8%)	
Ethnicity	Hispanic	3 (13%)	2 (18%)	1 (8%)	0.58
	Non-Hispanic	21 (88%)	9 (82%)	12 (92%)	
Reading level	4–6	1 (4%)	0 (0%)	1 (8%)	0.10
	7–8	12 (50%)	8 (73%)	4 (31%)	
	9+	11 (46%)	3 (27%)	8 (62%)	
Medication pickup assistance	Patients who had assistance picking up medication	18 (75%)	8 (73%)	10 (77%)	

*Number of individuals who had assistance picking up medication out of 24 total participants*.

Data were collected for 24 participants for visit 1, and 13 participants for visit 2 in this study. Eight participants in the interventional arm attended visit 2, but the psychological questions and the blood test were missed for one participant. Of the 12 research participants who did not complete visit 2, six had scheduled appointments and did not show and six could not be reached. There were 13 research participants who did not show up to their third visit because 8 could not be reached and 5 did not show.

The MPR was higher in the interventional group (0.95) than the control group (0.85) (see Table 2). The percentage of people who were adherent was numerically higher in the interventional group than the control (85 versus 64%), but the study was designed as a pilot and not for statistical significance. Evaluation of MPR was performed to see if there were differences by who did and did not drop out of the study. The MPR for the research participant in the interventional arm that appeared for visit 2 had a higher MPR (Table 2).

**Table 2:** **The medication possession ratio (MPR) of the control and interventional group, and the percentage of patients who has MPR <80%**.

MPR			
	Control (*n* = 11)	My Interventional Drug-Eluting Stent Educational App (MyIdea) (*n* = 13)	*P*-value
MPR (mean ± SD)	0.85 ± 0.24	0.95 ± 0.22	0.27
Poor adherence	36%	15%	0.36
**MPR for all consented participants**			**MPR (mean, SD)**
Control	Dropped out at visit 2		0.86 (0.30)
	Did not drop out at visit 2		0.84 (0.20)
MyIdea	Dropped out at visit 2		0.87 (0.28)
Did not drop out at visit 2			1.03 (0.13)

*P values were obtained using independent t-test and Fisher’s test. MPR for all consented participants: MPR by drop out at visit 2. Numbers in parentheses are SD. The MPR is greater than one due to the impact of filling the medication a few days early increasing the sum of day’s supply*.

Examining the two groups for differences revealed the control group was significantly more anxious at 8.8 from the HADS compared to the intervention group 4.2 (see Table 3). However, on the emotional scales of the SF-36, both groups appear to be about the same (see Table 3).

**Table 3:** **Baseline: the Hospital Anxiety and Depression Scale (HADS) scores and the SF-36 for both groups at visit 1**.

Psychometric data			
**HADS**	**Control (*n* = 11)**	**My Interventional Drug-Eluting Stent Educational App (MyIdea) (*n* = 13)**	***P-*value**
Anxiety	8.8 (5.2)	4.2 (3.4)	0.02
Depression	5.4 (2.4)	4.8 (2.2)	0.54
**SF-36 scale**	**Control (*n* = 11)**	**MyIdea (*n* = 13)**	***P-*value**
Physical function	41.4 (32.6)	44.2 (28.3)	0.82
Role limitation (physical)	31.8 (40.5)	42.3 (35.9)	0.51
Role limitation (emotional)	75.8 (42.4)	59.0 (43.4)	0.35
Fatigue	42.3 (21.3)	49.2 (26.2)	0.48
Emotional well-being	72.7 (17.3)	81.5 (17.3)	0.23
Social function	60.2 (30.0)	55.8 (34.5)	0.74
Pain	53.9 (32.7)	53.5 (29.6)	0.98
General health	50.0 (19.0)	47.3 (18.7)	0.73

*P-values were obtained using independent t-tests. HADS scale: 0 corresponds to low depression and anxiety and 10 corresponds to high depression and anxiety. Comparison of SF-36 results between the two groups. Due to high attrition rates for visit 2 and 3, group comparisons are not shown for those visits. Numbers in parentheses are SDs*.

When evaluating the biological measure of medication adherence between the two groups, the ARUXPRU score for visits 2 and 3 was lower for MyIDEA (Table S1 in Supplementary Material). An ARU and PRU value of 1 would indicate good adherence and 0 would be stopping the medication, with a value between the 0 and 1 related to the blood values of the Verifynow ARU and PRU tests. Due to limited follow-up by the research participants’ additional conclusions from these data were not warranted. In evaluating the beliefs about medication adherence between the two groups, the Morsiky-8 adherence questionnaire was approximately equivalent between the two groups (Table S2 in Supplementary Material).

For the individuals randomized to the interventional arm, we calculated the average time spent on the MyIDEA application for the first visit was approximately 20:42 min (SD = 8:26) and approximately 19:21 min (SD = 9:47) for the second visit. The average first visit time for all participants who appeared for their second visit was 13:19 min (SD = 1:35).

During visit 1, seven participants recorded that they related to Frank’s story (financial challenges), four for Eva (side effects) and one for Heather (visiting family) and one unrecorded. In visit 2, five participants related to Eva’s story, two Frank’s, one did not respond. A small increase knowledge was demonstrated in the interventional arm at visit 2 compared to the control group (Table 4).

**Table 4:** **Retention questionnaire showing the number of correct answers per visit for all participants**.

Retention and knowledge questionnaire				
	Interventional group		Control group	
	Visit 1	Visit 2	Visit 1	Visit 2
Average	7.53 (1.45)	8.33 (1.70)	7.50 (1.12)	7.50 (0.87)

## Discussion

This phase 1 pilot study focused on acceptability and usage of MyIDEA, a tailored, multi-educational style electronic tablet-based application for patient education. This study showed its potential and some of the challenges of conducting studies in the target population. The positive improvement in MPR for the intervention group compared to the control arm is promising, but further study is needed to determine if the effect measured is reproducible and sustained for a year, the duration of the therapy. Missing data from missed visits were substantial in this small study and indicate that additional resources will be needed to assist participants to complete study visits, perhaps with home visits or data collection over telephone.

### Medication Possession Ratio

In examining the differences in MPR, we evaluated the MPR in each arm by attendance at the second visit. Research participants in the interventional arm who attended the second visit had a higher MPR than those who did not attend visit two. This trend was not seen in the control group (Table 2). The ARU calculation in the interventional arm is less than the control arm. ARU calculation is dependent on attendance at visit two. Additional study is required to measure medication adherence for over the counter medication without prescription refills for ARU values. Factors such as automatic refills and 3-month prescription refills can also inflate MPR values. In addition, access to medication is not the same as taking the medication. In a prior study in the Veterans Affairs Hospital, a block away from where this study was conducted, 20.3% of patients were non-adherent (40) However, in the VA population, most of the medication is provided for little to no cost.

### MyIDEA Utilization

Analysis of the interventional arm about the usage of the MyIDEA program reveals in which sections of the program the patients spent the most time. We were able to use data tailored to each participant to augment the data provided by the clinicians about the procedure to show patients how-to problem solve issues such as monetary restrictions, transportation issues, and family support. The participants spent more time on the patient stories in their first visit than in their second visit, perhaps because they knew what to expect in visit 2.

The story patients most related to was Frank’s story. Frank’s story was related to monetary issues that kept him from getting his medication. This is consistent with the low-resource patient population served by the recruitment institution. The ways to help problem solve this issue was to rely on family and friends, have a good support system and always ask for help or talk to the physician about monetary restrictions.

### Comparison to Medication Interventions

Indeed, there are many mobile health developments that have recently hit the market, but these products and applications merely focus on improving adherence without really targeting the root of the problem in the first place. MyIDEA aims to intervene before the patient begins their prescription regimen, whereas other solutions only begin their intervention after the patient begins their medication. For example, GlowCap© uses a modified prescription bottle cap to remind users when to take their pills^2^. While the company states that they target the adherence issue of poor education, they only do so by weekly emails that are sent to the user after they begin using the product. However, there is no guarantee that users will thoroughly look through the email or even open it, which limits this aspect of the educational engagement of GlowCap. Similar approaches to solving adherence include the Kraken Medication Management system©^3^. This system tries fixing the problem by raising adherence rates but does not target poor education with medication adherence. Even smartphone applications, such as Mango Health, are trying to target the problem of poor adherence^4^. The app works by sending reminders to users and rewarding them with incentives if they take their medication. However, this system can be easily exploited as patients can say they are taking their medication without doing so and receive the rewards. Thus, MyIDEA takes a unique approach in that it is targeting the problem from the onset. MyIDEA aims to improve patient education before beginning a medication and engages participants without the requirement of a smartphone. MyIDEA, in other words, aims to improve medication adherence from a more preventative standpoint in comparison to current mobile health solutions.

### Limitations and Strengths

This is a single hospital with an enrollment of 24 research participants. One of the challenges with the MPR is that four research participants received a 90-day refill of their prescription, which when following research participants for 3 months would ensure a high MPR. Of the four participants, three of them were in the interventional group and one was in the control group. Response bias in the psychological tools is possible since the research participants would not want to let their physician down.

### Implications for Theory, Research, and Practice

Customer interactions with Amazon, Google, and all other technology are customized based on prior interactions with these companies. Customization has been driving innovation in many fields outside of medicine and is user friendly with an easy to understand format. This same idea can be transferred to the field of medicine for an easy-to-use learning application. The Kolb’s experiential learning theory is flexible enough for novel interventions that combine data recorded by clinicians, dynamic visualizations, and interactions with mobile technology. In this study, we use this theory to improve medication adherence. Participants are able to work with their own information in a user friendly format. In patient education, we should aim to integrate and customize the education to existing data within the Electronic Health Record to meet the unique needs of the patient condition. This customization will cater to different forms of learning that is suitable to most users. Additional research with MyIDEA and other customized educational tools is needed to evaluate if impact is similar.

## Conclusion

Future studies should focus on whether MyIDEA is creating a positive difference in medication drug adherence compared with treatment as usual. There is an ongoing discussion about non-inferiority of 6- versus 12-month DAPT with respect to the primary end point target lesion failure in a cohort of patients undergoing DES (41). There is currently not a definitive answer about differences between lengths of DAPT treatment, but all recommendations are dependent on the patient adherence, which MyIDEA has demonstrated to improve. Results from this study will enable new and effective educational programs to increase medication adherence as well as patient knowledge about the procedure that was performed on each participant. From the data obtained, we observed that the patients spent a larger percentage of time on patient stories, focusing on problem solving issues and critically thinking about solutions. Patient-centered health information used with an educational application is proving to be useful and progressive toward increasing drug adherence for patients. mHealth is promising but the technology needs to be developed to address the causes of poor medication adherence and not just technology to measure adherence. In the future, educational applications with the same concept as MyIDEA pertaining to other areas of medicine may also prove to be an effective way to engage patients.

## Ethics Statement

The University of Illinois at Chicago Institutional Review Board approved this study. All the participants had consented to the research project.

## Author Contributions

All the authors (VS, AD, CD, VG, BW, JF, MB, JF, AS, ZZ, YY, DW, and AB) have contributed to the design and analysis of the study, have critically review the manuscript, have reviewed the final manuscript before submission, and agree to the integrity of the manuscript.

## Conflict of Interest Statement

The authors declare that the research was conducted in the absence of any commercial or financial relationships that could be construed as a potential conflict of interest. The reviewer NG and handling Editor declared their shared affiliation, and the handling Editor states that the process nevertheless met the standards of a fair and objective review.

## Footnotes

^1^American College of Cardiology. *NCDR Elements and Definitions*. (2008). Available from: http://cvquality.acc.org/~/media/QII/NCDR/Data%20Collection%20Forms/CathPCI%20Registry_DataCollectionForm.ashx.

^2^Glow Caps. (2015). Available from: http://www.vitality.net/research.html.

^3^Kraken. (2015). Available from: http://www.impakhealth.com/medication-monitoring.

^4^Mango Health. (2015). Available from: https://www.mangohealth.com/about.html.
