# Supplementary material for: Patient-Centered Tablet Application for Improving Medication Adherence after a Drug-Eluting Stent
Source: Front Public Health. 2016 Dec 12;4:272. doi: 10.3389/fpubh.2016.00272 (PMC5149519; doi:10.3389/fpubh.2016.00272)
Supplement: Supplementary file 1 [file table_1.docx]

Supplementary Material

**Patient Centered Tablet Application for improving medication adherence after a Drug Eluting Stent**

**Vicki Shah^1^, BS, Anandu Dileep^1^, Carolyn Dickens, APN^2,3^, Vicki Groo, PharmD ^3,4^, Betty Welland^5^, Jerry Field^5^, Matthew Baumann^5^, Jose D Flores Jr^5^, Adhir Shroff^3^, Zhongsheng Zhao, PhD^2^, Yingwei Yao, PhD^6^, Diana J. Wilkie^6^, PhD, Andrew D. Boyd, MD^1^***

*** Correspondence:** Andrew D. Boyd, MD: boyda@uic.edu

# Supplementary Tables

**ARU, PRU**

|  | Visit 1 | | | Visit 2 | | Visit 3 | |
| --- | --- | --- | --- | --- | --- | --- | --- |
|  | Control (n=11) | MyIdea (n=13) | p | Control (n=6) | MyIdea (n=7) | Control (n=4) | MyIdea (n=6) |
| Raw ARU | 482 (90) | 467 (78) | .69 | 413 (55) | 484 (76) | 382 (32) | 462 (58) |
| Raw PRU | 155 (126) | 160 (102) | .92 | 164 (77) | 136 (115) | 182 (34) | 188 (110) |
| ARU score | N/A | N/A | N/A | 0.97 (0.04) | 0.89 (0.21) | 0.97 (0.03) | 0.90 (0.13) |
| PRU score | N/A | N/A | N/A | 0.96 (0.06) | 0.99 (0.02) | 0.93 (0.07) | 0.91 (0.08) |
| ARUxPRU | N/A | N/A | N/A | 0.93 (0.07) | 0.88 (0.22) | 0.91 (0.07) | 0.81 (0.11) |

Online Table 1. Aspirin Reactive Units (ARU), and Plavix Reactive Units (PRU) Numbers in parentheses are standard deviation

**Morisky Data**

|  | Visit 2 | | Visit 3 | |
| --- | --- | --- | --- | --- |
|  | Control (n=6) | myIDEA (n=7) | Control (n=3) | myIDEA (n=5) |
| Average | 2.1 | 1.5 | 2.0 | 2.2 |
| Standard Deviation | 1.0 | 1.4 | 1.0 | 1.2 |

Online Table 2. Morisky Data for Control vs. Interventional group.

Scale:

0 – High Adherence

1-2 – Medium Adherence

3-8 – Low Adherence

**Follow-up hospitalizations**

|  | **Control Group** | **Interventional Group** |
| --- | --- | --- |
| Non-associated cardiac hospitalizations | 5 (other) | 4 (other) |
| Associated cardiac hospitalizations | 1 (chest pain) | 2 (chest pain, scheduled cath) |

Online table 3: Follow-up hospitalizations and reasons for them
